# Attenuation of Mouse Hepatitis Virus by Deletion of the LLRKxGxKG Region of Nsp1

**DOI:** 10.1371/journal.pone.0061166

**Published:** 2013-04-08

**Authors:** Lin Lei, Sun Ying, Luo Baojun, Yang Yi, He Xiang, Su Wenli, Sun Zounan, Guo Deyin, Zhu Qingyu, Liu Jingmei, Chang Guohui

**Affiliations:** 1 State Key Laboratory of Pathogen and Biosecurity, Institute of Epidemiology and Microbiology, Academy of Millitary Medical Sciences, Beijing, China; 2 Institute of Disease Prevention and Control, Academy of Military Medical Sciences, Beijing, China; 3 State Key Laboratory of Virology and The Modern Virology Research Centre, College of Life Sciences, Wuhan University, Wuhan, China; The University of Hong Kong, China

## Abstract

Coronaviruses are a family of large positive-sense RNA viruses that are responsible for a wide range of important veterinary and human diseases. Nsp1 has been shown to have an important role in the pathogenetic mechanisms of coronaviruses *in vivo*. To assess the function of a relatively conserved domain (LLRKxGxKG) of MHV nsp1, a mutant virus, MHV-nsp1-27D, with a 27 nts (LLRKxGxKG) deletion in nsp1, was constructed using a reverse genetic system with a vaccinia virus vector. The mutant virus had similar growth kinetics to MHV-A59 wild-type virus in 17CI-1 cells, but was highly attenuated *in vivo*. Moreover, the mutant virus completely protected C57BL/6 mice from a lethal MHV-A59 challenge. To further analyze the mechanism of the attenuation of the mutant virus, changes in reporter gene expression were measured in nsp1- or nsp1-27D-expressing cells; the results showed that nsp1 inhibited reporter gene expression controlled by different promoters, but that this inhibition was reduced for nsp1-27D. The research *in vivo* and *in vitro* suggests that the LLRKxGxKG region of nsp1 may play an important role in this process.

## Introduction

Coronaviruses (CoV) are enveloped, positive-stranded RNA viruses of the order *Nidovirales*, family *Coronaviridae*, and subfamily *Coronavirinae* [1]. Coronaviruses infect many species of animal including humans, and can cause severe disease in livestock animals that results in high economic losses. In 2002–2003, the appearance of severe acute respiratory syndrome (SARS), caused by a formerly unknown coronavirus (SARS-CoV) [2]–[4], renewed the interest in this group of viruses.

Coronaviruses are divided into three groups. Group I viruses, including human coronavirus 229E (HCoV-229E), and Group II viruses, such as mouse hepatitis virus (MHV) and SARS, infect mammals, whereas group III viruses, such as infectious bronchitis virus (IBV), infect avian species. Coronavirus genomes are extraordinarily large, with sizes ranging from 27 to 32 kilobases (kb). One-third of the genome consists of the structural proteins, spike (S), envelope (E), matrix (M) and nucleocapsid (N), as well as accessory proteins specific to different strains. Approximately two-thirds of the genome encodes, in a single open reading frame (ORF), non-structural replicase proteins (nsp) that are involved in viral RNA synthesis. Many of the coronavirus nsp proteins have been shown, or are predicted, to have enzymatic functions, including papain-like cysteine proteinases (nsp3), 3C-like cysteine proteinase (nsp5), RNA-primase (nsp8), 2′O-MTase cofactor (nsp10), RNA-dependent RNA polymerase (nsp12), 5′-3′ helicase (nsp13), 3′-5′ exonuclease (nsp14), endoribonuclease (nsp15) and S-adenosyl-L-methionine-dependent (nucleoside-2′O)-methyltransferase (2′O-MTase, nsp16) and RNA cap N7-methyltransferase activity (nsp14) [5]–[9]. Nsp1 is the N-terminal cleavage product of the replicase polyprotein and is the first mature viral protein expressed in the host cell cytoplasm. Deletion of the nsp1-coding region in infectious clones of MHV yielded viruses that were unable to productively infect cultured cells. Furthermore, exogenous expression of MHV nsp1 in mammalian cells arrested the cell cycle in the G0/G1 phase and inhibited cell proliferation [10], [11]. SARS-CoV nsp1 protein induces degradation of host mRNA and suppression of translation both in nsp1-expressing cells and in SARS-CoV-infected cells [12]. *In vivo* studies have suggested that SARS-CoV nsp1 may counteract the host innate immune responses, thereby providing a survival advantage for the virus [13], [14]. Overall, these observations indicate that nsp1 may participate in multiple stages of the coronavirus life cycle, and implicate this protein as a potentially important virulence factor.

**Figure 1:**
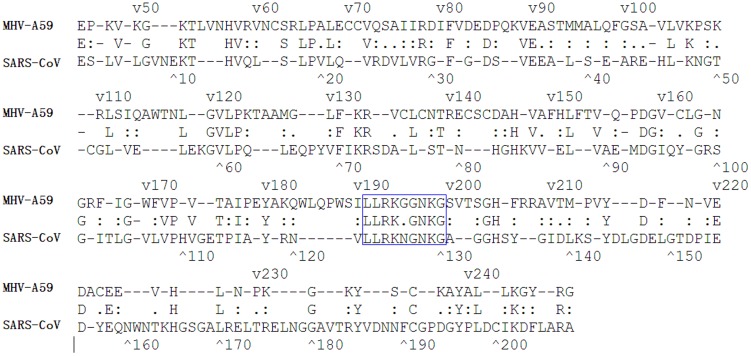
Sequence alignment of nsp1 from SARS-CoV and MHV-A59. The conserved amino acids are indicated in the middle line. A region (LLRKxGxKG: amino acids 191–199) in nsp1 that is conserved in different coronaviruses, such as MHV and SARS-CoV, is shown by the blue box.

Nsp1 sequences are divergent among the different coronaviruses, and this has made it challenging to identify the functional domains of nsp1. Sequence alignment has revealed that there is a region (LLRKxGxKG: amino acids 191–199) in nsp1 that is conserved in various coronaviruses, such as MHV and SARS-CoV (Figure 1) [15]. This conserved region may be an important functional domain, and hence a candidate region for deletion in order to generate an attenuated vaccine.

In order to identify the function of this conserved domain, we constructed a mutant MHV with deletion of the LLRKxGxKG region, using a reverse genetic system, and immunized C57BL/6 mice with the rescued mutant virus. By analyzing the growth kinetics of the mutant virus in cells, and assessing changes in mutant virus virulence *in vivo*, we hoped to characterize the role that the LLRKxGxKG region of MHV nsp1 plays in the pathogenetic mechanisms of this coronavirus.

## Results

### Generation of the MHV-nsp1-27D mutant virus, with deletion of the LLRKxGxKG region of nsp1

**Figure 2:**
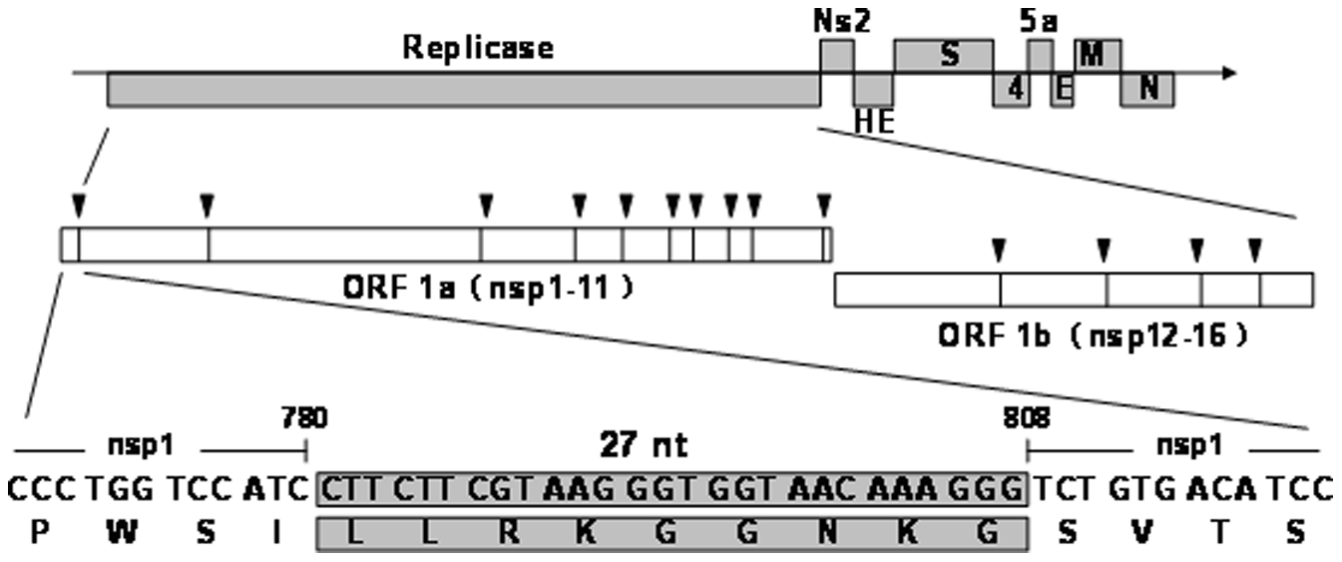
Organization of the MHV-A59 genome, and the relatively conserved region of nsp1 that was deleted in the mutated virus. The genome is a 29.7-kb positive-sense RNA molecule that is capped (dark line) and polyadenylated (arrow). Genes are indicated for the replicases (ORF 1a and ORF 1b; white), structural proteins [Spike (S), Envelope (E), membrane (M) and nucleocapsid (N); black] and accessory proteins. ORF 1b is accessed by a ribosomal frameshift in the nsp12 coding sequence. The ORF 1a/b polyprotein is translated directly from input genome RNA, and processed into 16 mature nsps by two virus-encoded proteinases. Nsps have predicted or demonstrated activities as described in the text. The residues (27 nt) putatively involved in the formation of the conserved region of MHV-A59 nsp1 are shaded; the nsp1-27D was constructed by deletion of these conserved amino acids.

**Figure 3:**
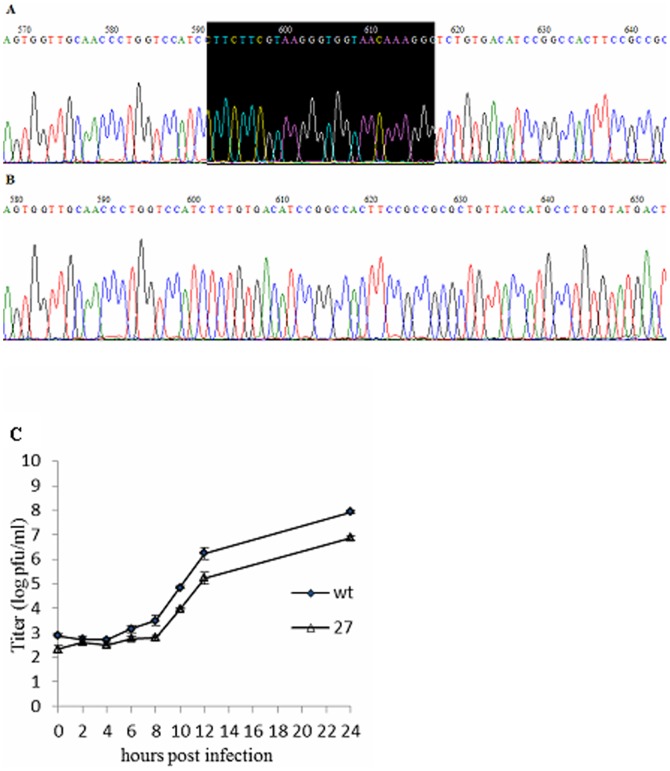
Sequence analysis and growth kinetics measurements of MHV-A59 and MHV-nsp1-27D. (A) WT MHV-A59 sequence. (B) MHV-nsp1-27D sequence, with deletion of nts 780–807 of nsp1. (C) Comparison of MHV-A59 and MHV-nsp1-27D growth. 17Cl-1 cells were infected with MHV-A59 or MHV-nsp1-27D at an MOI of 1 pfu/cell. Samples of culture medium were obtained at 0, 2, 4, 6, 8, 10, 12 and 24 h p.i., and viral titers were determined by plaque assay. Each time point indicates the mean titer and standard deviation obtained from a triplicate series of infections.

To assess the role of the LLRKxGxKG region of nsp1 in the context of viral replication, we constructed a recombinant virus, MHV-nsp1-27D, which encoded the MHV nsp1 with deletion of the LLRKxGxKG region (27 nts between nts 780–808) (Figure 2). The growth kinetics of both wild-type (WT) MHV-A59 and mutant viruses were determined by infecting murine 17Cl-1 cells with a multiplicity of infection (MOI) of 1. The plaque morphology of MHV-nsp1-27D was the same as that of WT MHV-A59 (data not shown). Both mutant and WT viruses began to replicate at 8 h post-infection (p.i.), and reached maximal titers at 12 h p.i. The maximal titer of MHV-nsp1-27D was about 5×10^5^ pfu/ml, a little lower than that of WT MHV-A59 (1×10^6^ pfu/ml). Viral growth and the peak titer of MHV-nsp1-27D were different from those of WT MHV-A59 (Figure 3C). To assess the stability of the recombinant virus, the nsp1-coding region of MHV-nsp1-27D was sequenced after five passages in tissue culture; sequence analysis showed that no nucleotide changes were detected (Figure 3A, B).

### The mutant virus MHV-nsp1-27D, with deletion of the LLRKxGxKG region of nsp1, shows reduced virulence in mice

**Figure 4:**
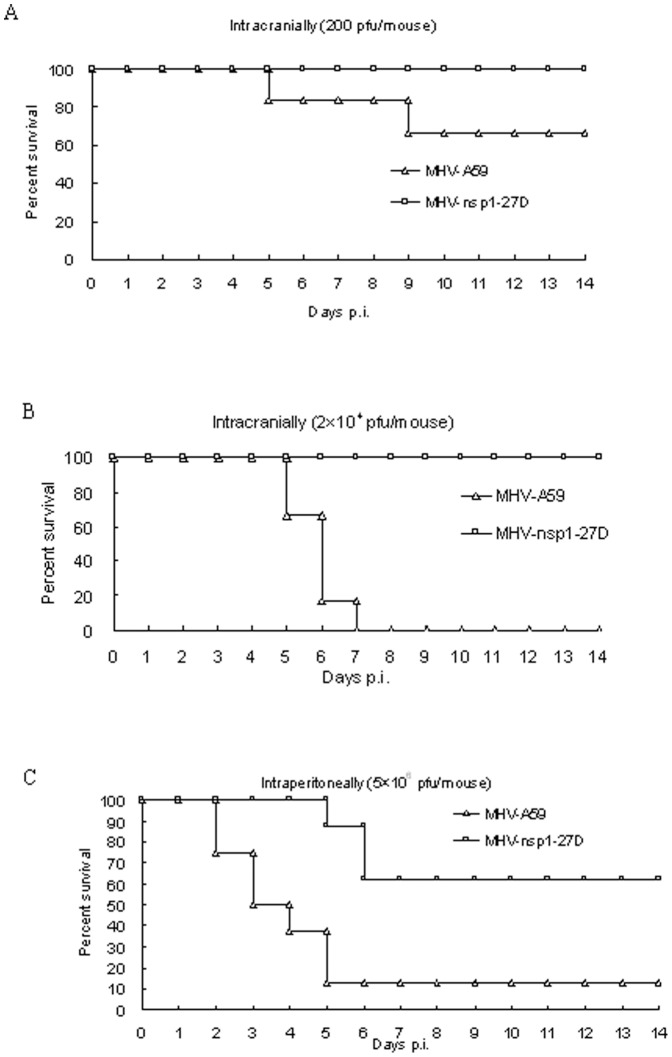
MHV-nsp1-27D is highly attenuated in mice. Groups of mice were infected intracranially (A, B) or intraperitoneally (C) with the indicated dose of MHV-nsp1-27D or MHV-A59. The survival rates of mice were monitored daily. For mice infected intraperitoneally with 5×10^6^ pfu of MHV-nsp1-27D or MHV-A59 (C), the animal weight was monitored daily. Each time point represents the mean data from at least five individual mice.

To evaluate the importance of the LLRKxGxKG region for virus virulence *in vivo*, C57BL/6 mice (n = 6 or n = 8)were infected intracranially (200 or 2×10^4^ pfu/mouse) or intraperitoneally (2×10^6^ pfu/mouse) with either MHV-nsp1-27D or MHV-A59. All mice infected with MHV-nsp1-27D by the intracranial route survived the 14-day observation period (Figure 4A, B). In contrast, mice infected intracranially with MHV-A59 began to die at day 5 p.i.: 33% (2 of 6) of the mice infected with virus at 200 pfu/mouse died on or before day 9 p.i., and all the mice (6 of 6) infected with virus at 2×10^4^ pfu/mouse died on or before day 7 p.i.

Mice infected by the intraperitoneal route with a high virus dose (2×10^6^ pfu/mouse) were found to have a lower average weight during the 14-day observation period, compared with mock-infected mice. Mice infected with MHV-A59 began to die at day 2 p.i., and by day 5 p.i., 87.5% (7 of 8) of the infected mice had died. In contrast, mice infected with MHV-nsp1-27D began to die at day 5 p.i., which was delayed when compared with mice infected with MHV-A59; in addition, 62.5% (5 of 8) of the infected mice survived the 14-day observation period (Figure 4C).

**Figure 5:**
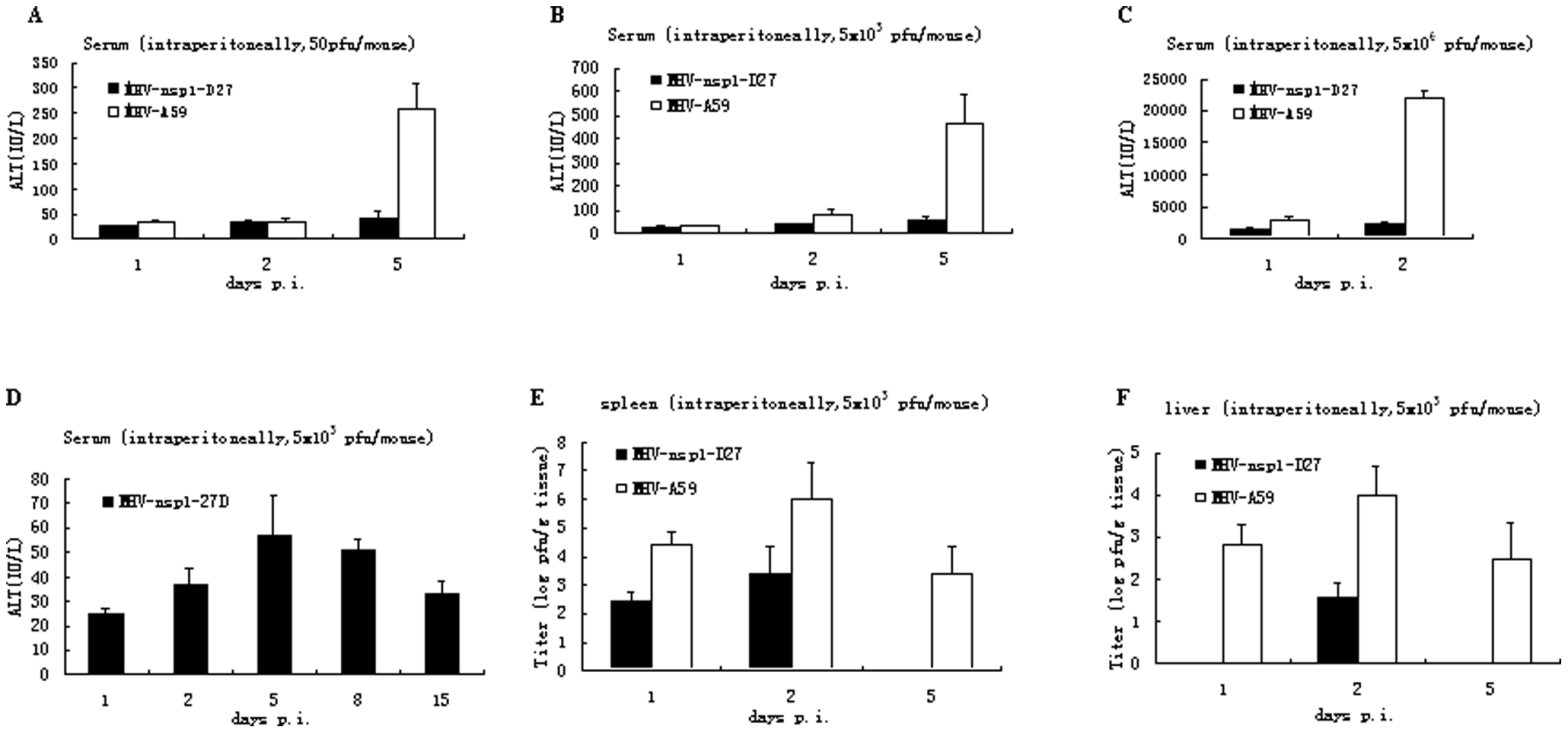
MHV-nsp1-27D is highly attenuated *in vivo*. C57BL/6 mice were infected intraperitoneally with the indicated dose of MHV-nsp1-27D or MHV-A59. Serum ALT values (A, B, C, D) were measured at the indicated time points. Virus titers in the liver (E) and spleen (F) were determined at the indicated time points.

MHV-A59 is a hepatotropic and neurotropic virus that can cause acute hepatitis and encephalitis in mice. Hepatitis is the first clinical sign of disease after MHV infection, and is accompanied by elevated levels of serum liver enzymes, such that the serum ALT level may be used as a marker for MHV-induced acute hepatitis [16], [17]. To further evaluate the effect of deletion of the LLRKxGxKG region on virus replication and pathology *in vivo*, C57BL/6 mice were infected intraperitoneally with either MHV-nsp1-27D or MHV-A59 at concentrations of 50, 5×10^3^ or 5×10^6^ pfu/mouse, and serum ALT levels were quantified at days 1, 2 and 5 p.i. (Figure 5A, B, C). The results showed that in mice infected with MHV-A59, serum ALT levels increased at day 5 from 50 to 260 IU/L with virus at 50 pfu/mouse, and from 20 to 500 IU/L with virus at 5×10^3^ pfu/mouse; for virus at 5×10^6^ pfu/mouse, the serum ALT level increased dramatically to 24000 IU/L at day 2 p.i. In contrast, serum ALT levels were much lower in mice infected with MHV-nsp1-27D, irrespective of the viral dose, and only minor differences were observed in serum ALT levels between the various time points studied. This observation correlated well with the lower virus titers found in the liver following infection with MHV-nsp1-27D.

To exclude the possibility that a reduced replicative capacity of the mutant virus may have resulted in a delayed hepatitis, ALT levels were also measured for a longer period of time (14 days) in mice infected intraperitoneally with 5×10^3^ pfu/mouse MHV-nsp1-27D. We observed that from day 5 onwards, there was a decline in ALT levels; at no time points was ALT elevated to a level indicative of hepatitis (Figure 5D). It therefore appears that MHV-nsp1-27D is not capable of inducing hepatitis in mice significantly, in contrast to MHV-A59.

For mice infected intraperitoneally with 5.0×10^3^ pfu/mouse MHV-A59 or MHV-nsp1-27D, the viral titers in the spleen and liver were quantified by plaque assay (Figure 5E, F). Both viruses replicated in the spleen and liver, but MHV-nsp1-27D titers were consistently lower than those of MHV-A59. Compared with the MHV-A59 virus, MHV-nsp1-27D replicated later (day 2 p.i.) in the liver, and was cleared earlier (day 5 p.i.) in the liver and spleen.

**Figure 6:**
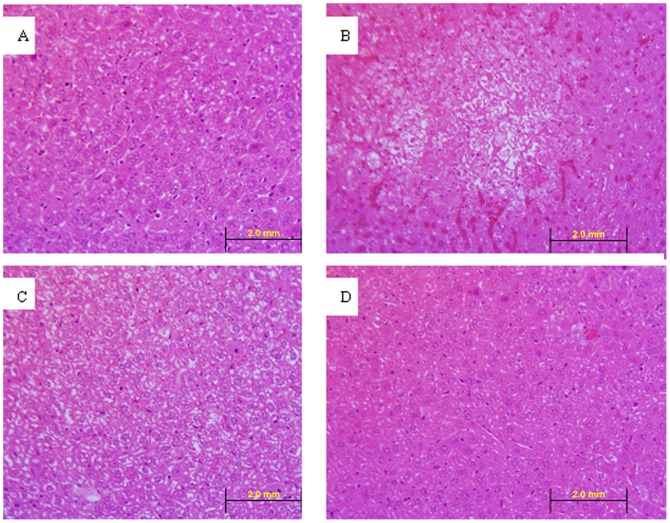
Hematoxylin and eosin staining of liver sections (40×). A: Liver section from a normal mice. B: Liver section from a mouse of the MHV-WT group, on day 5 (intra-abdominal infection, 5×10^3^ pfu/animal). C: Liver section from a mouse of the MHV-nsp1-27D group, on day 8 (intra-abdominal infection, 5×10^3^ pfu/animal). D: Liver section from a mouse of the MHV-nsp1-27D group on day 15 (intra-abdominal infection, 5×10^3^ pfu/animal). The liver of the MHV-WT infected mice showed obvious lesions on day 5. The hepatocytes in the center of the lesion were fibroblast-like and without nuclei. In MHV-nsp1-27D infected mice, the hepatocytes showed unclear borderlines and increased gaps on day 8, which was restored to a normal state on day 15.

To confirm the absence of a delayed hepatitis in animals infected with MHV-nsp1-27D, histological observations were made on liver slices stained with hematoxylin and eosin. In MHV-nsp1-27D infected mice, the hepatocytes showed unclear borderlines and increased gaps on day 8, but this was restored to a normal state on day 15 (Figure 6). Hence, no evidence of hepatic damage was evident at this time point.

Collectively, these data demonstrate that MHV-nsp1-27D is strongly attenuated *in vivo*, but retains the ability to replicate in secondary lymphoid organs, such as the spleen and liver.

### Immunization with the MHV-nsp1-27D mutant virus protects mice against challenge with WT MHV-A59 virus

**Figure 7:**
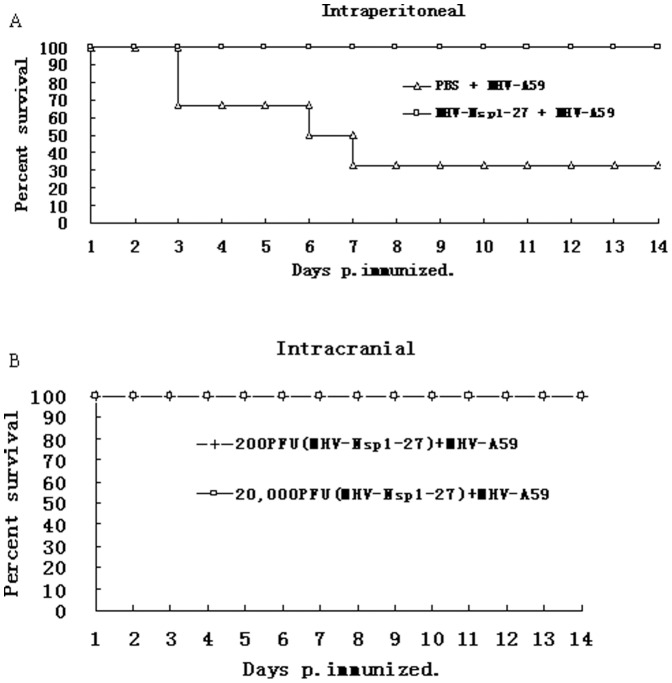
MHV-Nsp1-27D protects mice from challenge with WT MHV-A59. Groups of C57BL/6 mice (n = 6) were immunized (A) intraperitoneally (5×10^3^ pfu/mouse) or (B) intracranially (200 pfu or 2×10^4^ pfu/mouse) with MHV-nsp1-27D, or treated with PBS (A). 14 days p.i., mice were challenged with 5×10^6^ (A) or 2×10^4^ (B) pfu/mouse of WT MHV-A59. The survival of mice was monitored daily.

Phenotypic analysis of MHV-nsp1-27D revealed a number of features advantageous for development as a live attenuated vaccine: MHV-nsp1-27D grows to high titers in cell culture, can replicate in secondary lymphoid organs, and is strongly attenuated *in vivo*. In order to determine whether the attenuated mutant virus, MHV-nsp1-27D, could confer protection against challenge with WT MHV-A59, groups of C57BL/6 mice were either immunized with MHV-nsp1-27D, or treated with phosphate-buffered saline (PBS) as a control. Two-weeks p.i., mice were challenged with WT MHV-A59 by the same route, and the mortality determined 14 days post-challenge (Figure 7). The results showed that 70% of the PBS-immunized mice died on or before day 7 after the MHV-A59 challenge. In contrast, intraperitoneal vaccination with 5.0×10^3^ pfu/mouse of the MHV-nsp1-27D mutant completely protected mice from the lethal dose (5×10^6^ pfu) of WT MHV-A59. For the intracranial route, vaccination with either 200 or 2×10^4^ pfu/mouse of the mutant virus MHV-nsp1-27D was sufficient to achieve complete protection against challenge with 2×10^4^ pfu/mouse of the WT MHV-A59 virus. These data indicate that the mutant virus may be used as an attenuated live vaccine.

### Deletion of the LLRKxGxKG region reduced the inhibition of MHV nsp1 on different promoters

**Figure 8:**
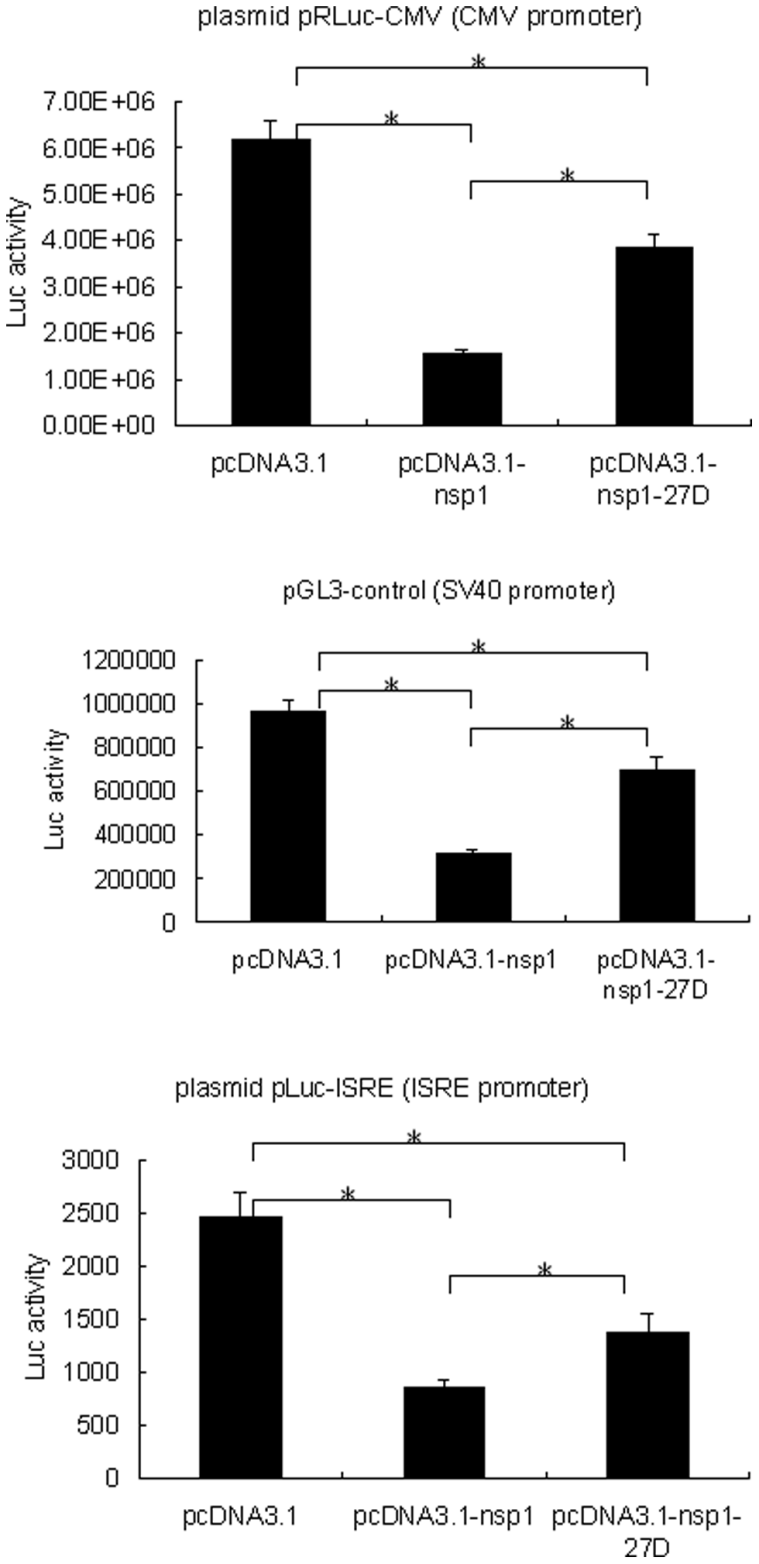
MHV nsp1 and nsp1-27D reduce the expression of reporter genes controlled by different promoters. BHK-21 cells were co-transfected with pRLuc-CMV (CMV promoter), pGL3-control (SV40 promoter) or pLuc-ISRE (ISRE promoter), and the indicated expression plasmids. 24 h post transfection, the luciferase activity was measured. The results represent the means of at least four independent experiments. Statistical analysis was performed using paired Student's t-test (* p<0.01).

Viruses have evolved a variety of mechanisms to evade the antiviral response of the host, such as directly antagonizing specific components of IFN-dependent signaling or IFN effector pathways. Some viruses indirectly antagonize the antiviral response by inhibiting the general mechanisms of host cell gene expression [18]–[20]. A recent report suggested that SARS-CoV nsp1 suppressed host cell gene expression, inducing generalized host cell mRNA degradation [12]. In order to reveal the potential role of the LLRKxGxKG region in the attenuation of the MHV-nsp1-27D mutant virus, we analyzed changes in reporter gene expression in cells expressing nsp1 or nsp1-27D. BHK-21 cells were co-transfected with different expression plasmids (pcDNA3.1, pcDNA3.1-nsp1 or pcDNA3.1-nsp1-27D) and reporter plasmids. The activity of the reporter gene, luciferase, was assessed; this was under the control of the CMV and SV40 promoters, and the interferon stimulated response element (ISRE). The results showed that co-expression of MHV-nsp1 significantly reduced the luciferase activity (Figure 8) by about 6-fold (P<0.01) for the CMV promoter, 3-fold (P<0.01) for the SV40 promoter and 3-fold (P<0.01) for the ISRE. By comparison, co-expression of MHV-nsp1-27D significantly decreased luciferase activity by about 2-fold (P<0.01) for the CMV promoter, 1.5-fold (P<0.01) for the SV40 promoter and 1.5-fold (P<0.01) for the ISRE. Our results indicate that MHV nsp1 can suppress host cell gene expression, but that deletion of the LLRKxGxKG region attenuates this effect. This further suggests that the conserved region, LLRKxGxKG, may play an important role in the suppression of host cell gene expression.

**Figure 9:**
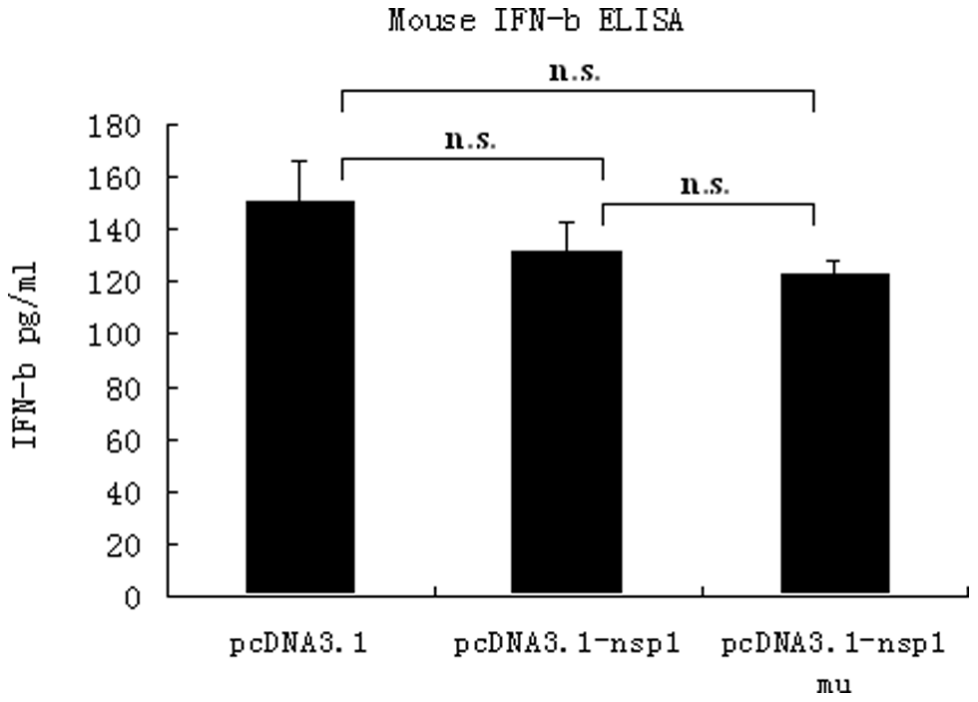
IFN-ß levels in L929 cells expressing either nsp1 or nsp1-27D. L929 cells were transiently transfected with the recombinant plasmids, pcDNA3.1-nsp1 or pcDNA3.1-nsp1 mu, and stimulated with NDV. The IFN-β level in the supernatant was determined using an ELISA kit. The IFN-β levels in the supernatant of cells transfected with pcDNA3.1-nsp1 and pcDNA3.1-nsp1 mu were decreased (130.8 pg/ml and 122.4 pg/ml) compared with cells transfected with control pcDNA3.1 (149.9 pg/ml). n.s., p>0.05.

In further experiments, the genes for nsp1 and nsp1-27D were transfected into L929 cells in order to determine their effects on IFN-ß expression. We found that both nsp1 and nsp1-27D were without significant effect on IFN-ß expression (Figure 9), implying that the functional consequences of deletion of the LLRKxGxKG region in nsp1 are not due to changes in IFN-ß levels.

## Discussion

Coronaviruses are a family of large, positive-sense RNA viruses that are responsible for a wide range of important veterinary (*e.g.* bovine coronavirus) and human (*e.g.* SARS-CoV, HCoV-OC43, HCoV-229E) diseases [21]–[24]. MHV is a group II coronavirus that has long been used as a tool for studying coronavirus biology and pathogenesis.

The N-terminal cleavage product of the polyprotein encoded by the replicase gene of MHV, nsp1 (p28), has been shown to have a role in pathogenesis *in vivo*. A mutant of MHV-A59, with a deletion of 99 nucleotides in the C-terminal portion of nsp1 (nts: 829–927), was found to replicate with similar kinetics and to a similar titer as WT virus *in vitro*; however, the nsp1 mutant was attenuated *in vivo*. Expression of SARS-CoV nsp1 (p20) has been shown to inhibit RNA expression regulated by an IFN-β promoter, but a mutant nsp1 (with deletion of the C-terminal 13 amino acids) was found to lack this action [25]–[26]. These observations illustrate that MHV-A59 nsp1 and SARS-CoV nsp1 have similar activities to inhibit cellular protein expression.

Nsp1 sequences are divergent among the different coronaviruses, and no sequence similarities between SARS-CoV nsp1 and group II nsp1 proteins have been identified using standard searching tools such as BLAST. Amino acid sequence alignment of SARS-CoV and MHV-A59 nsp1 showed a relatively conserved domain, LRKxGxKG (L191-G199). Furthermore, analysis of the structure of SARS nsp1 indicated that this domain might be a candidate domain for mRNA interaction. Since the amino acids deleted in this study were included in the region K124-L241, which has been reported to have no effect on virus replication and the stability of NSP1 protein [10], the deletion of LRKxGxKG region would not be expected to cause instability of NSP1.

The use of reverse genetics to construct recombinant viruses has enabled us to identify important virulence factors, and has facilitated the rational design of live attenuated viral vaccines. This study demonstrates that an unprecedented level of attenuation is achieved through deletion of the LLRKxGxKG region in MHV-A59 nsp1. Firstly, we found that survival of C57BL/6 mice infected with MHV-nsp1-27D was higher than that of mice infected with MHV-A59, irrespective of whether the route of infection was intracranial or intraperitoneal. Secondly, the results showed that MHV-nsp1-27D was unable to cause acute hepatitis, as evidenced by the much lower serum ALT levels than those seen in mice infected with MHV-A59. Thirdly, in pathological investigations designed to explore the importance of the LLRKxGxKG region for virus virulence *in vivo*, intraperitoneal infection of mice with MHV-A59 or MHV-nsp1-27D revealed that both viruses replicated in the spleen and liver, but that MHV-nsp1-27D titers were consistently lower than those of MHV-A59. Collectively, these data indicate that MHV-nsp1-27D is strongly attenuated *in vivo*, but retains the ability to replicate in secondary lymphoid organs. In addition, immunization with mutant virus (5000 pfu/mouse intraperitoneally or 200 pfu/mouse intracranially) was sufficient to protect C57BL/6 mice from subsequent challenge with an otherwise lethal dose of WT MHV-A59. This strongly suggests that the mutant virus, MHV-nsp1-27D, has potential for use as an attenuated live vaccine.

To further analyze the mechanisms by which deletion of the LLRKxGxKG region causes attenuation of the mutant virus, both MHV nsp1 and nsp1-27D were expressed in cultured cells. Although expression of MHV-nsp1 and nsp1-27D appeared to reduce the production of IFN-β induced by Newcastle disease virus (NDV), the differences between the control group and either the MHV-nsp1 or nsp1-27D groups were not significant. However, in further experiments assessing whether expression of MHV-nsp1 or nsp1-27D was able to influence reporter gene expression driven by the SV40 and CMV promoters, we found that inhibition of reporter gene expression was significantly reduced after deletion of the 27 nts. These observations support the notion that nsp1 has an antagonistic activity against IFN. However, this inhibitory action may not be a direct effect on IFN signaling, and may represent the result of removing the inhibitory action of nsp1 on host cell mRNA [12].

## Materials and Methods

### Mice and cells

Three- or 4-week-old C57BL/6 mice were purchased from Vital River Laboratory Animal Technology Co. Ltd. (Beijing, China). Groups of mice were maintained in individually ventilated cages in a bio-safety level 2 (BSL2) laboratory. Institutional guidelines for animal care and use were followed throughout the experiments.

17Cl-1 (mouse fibroblast), L929 (mouse fibrosarcoma), CV-1 (monkey kidney fibroblast), BHK-21 (baby hamster kidney) and HeLa-D980R (human cervical) cells were purchased from the European Collection of Cell Cultures (http://www.ecacc.org.uk/). 17Cl-1, L929 and HeLa-D980R cells were cultured at 37°C in Dulbecco's Modified Eagle's Medium (DMEM) supplemented with 5–10% fetal bovine serum (FBS), 100 U/ml penicillin, 100 µg/ml streptomycin and 5% tryptose phosphate broth (TPB). CV-1 and BHK-21 cells were cultured at 37°C in Minimal Essential Medium (MEM) supplemented with HEPES (25 mM), 5% FBS and antibiotics. The regulatable BHK-21 cell line, expressing the MHV-A59 nucleocapsid protein (BHK-MHV-N cells) under the control of the TET/ON system, was a kind gift from Prof. Stuart G. Siddell, University of Bristol, Bristol, UK; these cells were cultured as described previously [27]–[29].

### Construction of recombinant mutant viruses

Recombinant vaccinia virus inf-1 (vMHV-inf-1) DNA, which contains a cloned, full-length MHV-A59 cDNA sequence (GenBank accession number NC001846), was kindly provided by Prof. Stuart G. Siddell. MHV-A59 nsp1 (nts 1–951) was amplified by standard PCR techniques using vMHV-inf-1 DNA as a template, and nsp1-27D (nsp1 with deletion of nts 780–807) was amplified by overlap PCR. Mutant vaccinia viruses were based on the recombinant vaccinia virus, vMHV-inf-1, and generated using a reverse genetic system as described previously [28], [29]. Briefly, the vMHV-inf-1 nsp1 gene was first replaced by the *Escherichia coli* guanine-phosphoribosyl-transferase (GPT) gene, through vaccinia virus-mediated homologous recombination with the plasmid, pGPT-in-nsp1; this plasmid contains the GPT gene flanked to its left by MHV-A59 nts 1–446, and to its right by MHV-A59 nts 856–1310. GPT-positive clones were selected by three rounds of plaque purification on CV-1 cells, in the presence of xanthine, hypoxanthine and mycophenolic acid (GPT-positive selection). In a second round, the GPT gene was replaced by the nsp1-27D gene, and GPT-negative clones, vMHV-nsp1-27D, containing the mutant gene, were selected by three rounds of plaque purification on D980R cells in the presence of 6-thioguanine (GPT-negative selection). Further details of the cloning procedure, plasmid maps and sequences are available from the authors upon request.

Recombinant mutant MHV-nsp1-27D virus was rescued from cloned cDNA using purified, EagI-cleaved vaccinia virus vMHV-nsp1-27D DNA as a template for the transcription of RNA, which was electroporated into BHK-MHV-N cells as described previously [29]. Following electroporation, the transfected BHK-MHV-N cells were mixed with a four-fold excess of 17Cl-1 cells. At days 1 and 2 post-electroporation, tissue culture supernatants were taken, recombinant coronaviruses were plaque purified three times, and a single plaque was used to produce a virus stock. The identity of recombinant MHV-nsp1-27D was confirmed by sequence analysis.

### Viral growth assays

For determination of viral growth, 17Cl-1 cells were infected with MHV-A59 WT and mutant viruses at multiplicities of infection (MOI) of 1 to 5 pfu/ml. Following a 45-min adsorption, with rocking at room temperature (RT), the medium was aspirated, and cells were washed three times with PBS and then incubated with pre-warmed medium at 37°C. Aliquots of medium were collected from 1 to 24 h p.i., and virus titers were determined using a plaque assay, as described previously [29].

### Viral infections, and determination of viral titers and alanine aminotransferase (ALT) levels

Groups of 3–4-week-old C57BL/6 mice were injected intraperitoneally with the indicated pfu of WT MHV-A59 or mutant MHV-nsp1-27D. Mice were sacrificed at the indicated time points, and the livers and spleens were harvested from the infected animals and stored at −70°C. Viral titers were determined using a plaque assay. Blood was incubated at RT to allow coagulation to occur, and was then centrifuged to obtain serum; the serum was used for measurements of ALT levels using a Hitachi 747 autoanalyzer (http://www.hitachi.com/).

### Histological examination of liver specimens

Tissues were fixed in 4% formalin and embedded in paraffin. Sections were stained with hematoxylin and eosin. Images were acquired using an OLYMPUS BX61 microscope (OLYMPUS, http://www.olympus.com/) with a 40×/1.00 Oil Iris objective. Images were processed using Adobe Photoshop (Adobe Systems, http://www.adobe.com).

### Virulence *in vivo*

To evaluate the virulence *in vivo*, groups of C57BL/6 mice (n = 6 or 8) were infected with different doses of WT MHV-A59 or mutant MHV-nsp1-27D, via intracranial (30 µl) or intraperitoneal (500 µl) routes. Survival of the mice was monitored over a 14-day observation period. For protection tests, groups of C57BL/6 mice were infected with a low dose of mutant MHV-nsp1-27D, and two weeks later, the mice were challenged with a high dose (5×10^6^ pfu/mouse) of WT MHV-A59. The mice were monitored for clinical symptoms and death, up to day 14 post-challenge.

### Recombinant plasmids and *in vitro* reporter gene assay

NDV virus was grown in 10-day-old embryonated eggs, cultured and titered as described previously [30]. The amplified MHV-A59 nsp1 (nts 1–951) and nsp1-27D (nsp1 with deletion of nts 780–807) were sequenced and cloned into the expression plasmid, pcDNA3.1-myc-his-a (Invitrogen, Beijing, China). The reporter plasmid, p(-110-IFN-β)-CAT, was used for monitoring IFN-β promoter activation; this was kindly provided by Prof. Tom Maniatis (Harvard University, USA). The luciferase (Luc) reporter plasmid, pISRE-Luc (Stratagene, USA), was used to monitor activation of the interferon-stimulated response element (ISRE), and the reporter plasmids, pRLuc-CMV (Promega, Shanghai, China) and pGL3-control (Promega, Shanghai, China), were used to monitor the activities of the CMV and SV40 promoters, respectively.

For the reporter gene assay, sub-confluent cell monolayers in 12-well plates were transfected with different expression plasmids (pcDNA3.1, pcDNA3.1-nsp1 or pcDNA3.1-nsp1-27D) or co-transfected with different reporter plasmids. At 18 h post-transfection, the cells were induced with either NDV (16 HAU/well) or 2000 U/ml hIFN-α (Sigma, Shanghai, China), or were left un-induced. After a period of incubation, the chloramphenicol acetyltransferase (CAT) activities were measured using the CAT ELISA Kit (Roche, Basel, Switzerland); luciferase assays were performed using the Dual-Luciferase Reporter 1000 Assay System (Promega, Shanghai, China).

### Measurement of IFN-ß levels

Nsp1 and nsp1-27D genes were inserted into pcDNA3.1 vectors, and these were used to transfect L929 cells. After induction with NDV, the IFN-ß concentration in the supernatant of the cells was determined using an enzyme-linked immunosorbent assay (ELISA) kit.(PBL Biomedical Laboratories, http://www.interferonsource.com/)according to the manufacturers instructions.

### Statistical analysis

All statistical analyses were performed using Prism 4.0 software (GraphPad Software, http://www.graphpad.com/). Data were analyzed using the paired Student's t-test, with the assumption that the values followed a Gaussian distribution. A p-value of <0.05 was considered to be indicative of statistical significance.
