# Correction: Attenuation of Mouse Hepatitis Virus by Deletion of the LLRKxGxKG Region of Nsp1

**DOI:** 10.1371/journal.pone.0094740

**Published:** 2014-04-03

**Authors:** The PLOS ONE Staff

**Notice of Republication**

This article was republished on March 20, 2014, to replace confidential or copyrighted material. Please download this article again to view the corrected version.
